# Spatial Distribution of Road Traffic Accident at Hawassa City Administration, Ethiopia

**DOI:** 10.4314/ejhs.v31i4.14

**Published:** 2021-07

**Authors:** Shamenna AkliluToma, Bedane Ashenafi Senbeta, Ali Anteneh Bezabih

**Affiliations:** 1 Department of Statistics, College of Natural and Computational Sciences, Hawassa University, Hawassa, Ethiopia

**Keywords:** Hawassa, RTAs, clustering, hotspot, Getis-Ord Gi*, Moran's I

## Abstract

**Background:**

Globally, road traffic accidents (RTAs) are the leading killer of young people and are projected to be the 7^th^ leading cause of death by 2030. This study is aimed at analyzing the spatial distribution of road traffic accident and identifying hotspot areas across Kebeles (smallest administrative division in Ethiopia) of Hawassa city administration in Ethiopia.

**Method:**

Secondary data on daily traffic accident record from October 2013 to June 2018 was obtained from Hawassa city administration police department. The spatial clustering and hotspots identification were carried through Moran's I and Getis-Ord Gi* statistics. Data analysis was conducted using GeoDa 1.16.0.0 and ArcGIS 10.2 softwares.

**Results:**

Drivers within age group of 18–30 years, who were hired by private business owners and who had no driving license committed the highest number of traffic accidents. The majority of traffic accidents were caused due to careless driving, failure to give priority for pedestrian, high speed and driver failure to give priority for each other. In addition, about 82.01% of traffic accidents were recorded on asphalts road and 11.51% by gravel road. Spatial clustering of road traffic accidents for accidents occurred on gravel road and in sunny weather conditions found to be significant. Different hotspot areas were identified for gravel type of road and sunny weather condition.

**Conclusion:**

The concerned government bodies involved in policymaking are recommended to give special attention to young driver who were hired by private business owners. Interventions to mitigate the occurrence of traffic accident would take in to account the identified hotspot areas.

## Introduction

Road traffic accidents (RTAs) constitute a major global public health problem being a cause for the worldwide death of about 1.25 million people each year. RTAs are the leading cause of death among people aged 15 and 29 years and are predicted to become the seventh leading cause of death by 2030. Middle and low-income countries, owning only 54% of the world's vehicle, constitute about 90% of the world's fatalities on the roads. Nearly half of those dying on the world's roads are vulnerable road users who namely are pedestrians, cyclists and motorcyclist. RTA's cost most countries 3% of their gross domestic product (1).

RTAs affect not only the health of individuals but also their family members, as it can drive households in to poverty when they struggle to cope with the long term consequence of the event, including the cost of medical care and have also a huge strain on the national health systems (1, 2). Low and middle income countries share the highest burden of RTA were over 85% of fatalities and 90% of life year lost are reported (1–3).

According to World Health Organization (WHO) report, even though global RTA fatalities remain constant since 2007, the rate is increasing in many of developing countries (1). Africa faces the highest annual rate of RTA fatalities in the world and the problem is expected to increase in the next decades due to on-going rapid economic growth and increasing motorization in the continent (4,5).

It is reported that RTA remained neglected health problem in many developing countries and the health sector in developing countries has been slow to recognize RTA as a priority public health problem (1–3,6). Developing counties were successful in reducing incidence of RTAs through cost-effective interventions while many Sub Saharan African (SSA) counties were facing enormous road safety crisis (3,7,8).

In Ethiopia, which is one of least developed countries in the world, thousands of road users are reported to be killed each year, majority of whom were economically active population (2, 9). Rampant reckless driving behavior, poor road network, substandard road conditions, failure to enforce traffic lows and poor vehicle conditions are among major factors contributing to RTA's in Ethiopia (1–3,10,11).

A study conducted in Dilla town in Ethiopia by (12) indicated that RTA is the most common type of injury and it accounts for higher percentage of deaths among trauma patients. The rate of occurrence of RTAs in Ethiopia is rising from time to time and RTAs are becoming series public health problem. Rapid population growth, rapid motorization and increase in road network coupled with poor attitude and habits of road users put the country at higher risk of RTAs (10).

The magnitude of the effect of RTAs could be greatly reduced if preventive measures were taken in safety deficient geographical zones (11, 13). Geographical information systems (GIS) are a very important and comprehensive management tool for traffic safety. GIS-aided spatial analysis of traffic accident has always interested researchers and GIS users as they provide information on hazardous regions, hot spot and cold-spot identification (14, 15).

Moreover, exploratory spatial data analysis (ESDA) and spatial autoregressive models have brought new power to the analysis of spatial data, capturing the effects of redundant location information contained in this data. Their descriptive abilities better portray observed data and they offer more fully developed nomothetic approach that in turn will support an increasing emphasis on geographically varying local statistics that fluctuate around their global parameter counterparts (16, 17).

The clustering of traffic accidents based on time and space always raises questions about the location and the reasons for that location (18–23). Most notably, spatial thinking helps to identify the patterns and suggest reasons for the pattern characteristics.

Spatial patterns of RTA at the local level (fixed spatial scale) in Africa, and particularly in Ethiopia, have not been well investigated or accurately defined. Such research is needed in developing dynamic and area-specific risk maps to identify locations and populations at highest risk for appropriate planning and implementation of preventive and control measures.

Having this background, this study is intended to explore spatial distribution of RTA across Kebeles (smallest administrative divisions in Ethiopia) in Hawassa City Administration (HCA), Southern Ethiopia using Exploratory Spatial Data Analysis (ESDA) technique.

## Methods

Description of the Study Area and Population: The study was conducted at Kebele level in HCA in Southern Ethiopia. Based on the 2007 Census conducted by the Ethiopian Central Statistics Authority (CSA), HCA has a total population of 124, 472 of whom 62,774 are men and 61,698 women (24).

**Study design**: Descriptive study design was adopted. The study was based on RTAs data obtained from 24 hours traffic police records for each Kebele of HCA police office. Police records contain the frequency of RTA, and information on each event including sociodemographic and economic characteristic of driver as well as the environmental conditions at the time of the crash and vehicular characteristics. The data aggregated from police department for each Kebeles of HCA from October 11, 2013 to June 08, 2018 was analyzed. Shape file was obtained from Finance and Economic development office of Southern Nations Nationalities and Peoples Regional State (SNNPRS). Data analysis is based on GeoDa 1.16.0.0and ArcGIS 10.2 software (25).

**Geospatial analysis**: Geospatial data was analyzed by geographic locations to evaluate geospatial distribution and evaluate areas with higher densities of occurrences (hotspots) of traffic accident rate at Kebele level. Exploratory spatial data analysis (ESDA) was applied through the use of GeoDa™ software version 1.16.0.0 (Spatial Analysis Laboratory, University of Illinois, Urbana Champaign, IL, USA) to determine measures of global spatial autocorrelation and local spatial autocorrelation. Moreover, for spatial visualization of the data, Local Indicators of Spatial Association (LISA) maps were presented (25,26).

**Spatial weight matrix**: The associations of neighborhood observations, defined for each location, can be expressed by spatial contiguity or a weight matrix W of order *n × n*, where n is the number of neighboring locations. The spatial wight matrices W_1_ and W_2_ are defined using Queen contiguity method of spatial weight matrix which defines neighbours such that if a portion of boundary (either edge or vertex) between two regios is shared, the corresponding element of spatial weight matrix W_ij_ is 1 and 0 otherwise (25–27).

$$W=\left(\begin{array}{cccccc} w_{11} & w_{12} & \cdot & \cdot & \cdot & w_{1n}\\ w_{21} & w_{22} & \cdot & \cdot & \cdot & w_{2n}\\ \cdot & \cdot & \; & \; & \; & \cdot \\ \cdot & \; & \cdot & \; & \; & \cdot \\ \cdot & \; & \; & \cdot & \; & \cdot \\ w_{n1} & w_{n2} & \cdot & \cdot & \cdot & w_{nn} \end{array}\right)$$

**Spatial autocorrelation**: Spatial correlation is the correlation between observations of as single variable solely attributable to their proximity in space. Spatial autocorrelation measurements and tests can be differentiated by the range or scale of analysis, as distinguished from global and local measures (25, 27). A global measure implies that all measurements in the matrix W are included in the spatial correlation calculation, producing a spatial autocorrelation value for any spatial weight matrix while local measures evaluate the autocorrelation associated with one particular area or a few area units rather than all of them (27).

Both measurements indicate the degree of spatial association of the data set. To evaluate the existence of spatial autocorrelation, we used Queen Contiguity matrix that allows for the measurement of non-random association between the value of a variable observed in a given geographical units and the value of variables observed in neighboring units (26, 28).

The Moran's I index calculates the spatial autocorrelation as a covariance from the product of the deviations from the mean (27). This index calculates the spatial association present in the data set within all locations under consideration. The global version of Moran's I index is calculated using the following formula:

$$I=\frac{\sum_{i=1}^{n}\sum_{j=1}^{n}W_{ij}(y_{i}-\bar{y})(y_{j}-\bar{y})}{\sum_{i=1}^{n}{\left(y_{i}-\bar{y}\right)}^{2}}$$

Assuming that *Z_i_* is an observation of random variables *Z_i_* whose distribution is normal, then Moran's I has approximately normal distribution with expected value and variance given by (26):

$$\begin{array}{l} E(I)=-\frac{1}{\left(n-1\right)}\\ Var(I)=\frac{n^{2}(n-1)W_{1}-n(n-1)W_{2}-2W_{0}}{(n+1){\left(n-1\right)}^{2}W_{0}} \end{array}$$

The Moran's index varies in a range of -1 to 1 where -1 means perfect dispersion, 0 represents random behavior and 1 means perfect association. For all given set of locations and an associated attribute, it evaluates whether the pattern expressed is clustered, dispersed, or random (29).

In this study, Moran's I index was used as a general test to see spatial clustering of annualized average incidence rate of RTA per 1000 persons. In spatial data analysis, our interest lies not only in determining whether the data as a whole exhibit spatial autocorrelation, but also in identifying the specific observations that exhibit spatial autocorrelation with their neighbors (26).

In his regard, global Moran's I index, as comprehensive measure, are useful to provide an indication of global grouping data, but such methods need to be complemented by local statistics (30).

The local version of Moran's I index and Getis-Ord Gi* statistics (30) are used in identifying spatial clusters of high values (hotspots) and of low values (cold spots).

Local version of Moran's I index for each observation measures the extent of significant spatial clustering of similar values around that

$$\begin{array}{l} E(G_{i}^{*})=\frac{\sum_{j=1}^{n}W_{ij}}{n}\\ Var(G_{i}^{*})=\frac{W_{i}^{*}(n-E(G_{i}^{*}))y_{i2}}{n^{2}(n-1)y_{i1}}\\ \text{Where }W_{i}^{*}=\sum_{j=1}^{n}W_{ij}\\ y_{i1}=\sum_{j=1}^{n}y_{j}n \end{array}$$

observation. The local version of Moran I statistic for each location is given by (26)

$$I_{i}=\frac{(y_{i}-\bar{y})\sum_{j=1}^{n}W_{ij}(y_{j}-\bar{y})}{\sum_{i=1}^{n}\frac{{\left(y_{i}-\bar{y}\right)}^{2}}{n}}$$

Where *n* the number of spatial locations, *W_ij_* is the element in the spatial weight matrix corresponding to the location pair *i,j* and *y_i_* and y*j* are the number of accidents for location pairs *i* and *j* with average number of accidents *ȳ*.

Positive values of *I_i_* mean that there are spatial clusters with similar values (high or low) of the variable under the study, whereas negative values mean that there are spatial clusters with dissimilar values of the variable in and between the areas and neighbors (27).

The Getis-Ord G^*^_i_ test statistic is given by (30):

$$G_{i}^{*}=\frac{\sum_{j=1}^{n}w_{ij}y_{j}-\left(\sum_{j=1}^{n}w_{ij}+w_{ii}\right)\bar{y}}{S{\left(\frac{\left[NS_{i}^{*}-{\left(\sum_{j=1}^{n}w_{ij}+w_{ii}\right)}^{2}\right]}{n-1}\right)}^{\frac{1}{2}}}$$

Where *w_ij_* represents the elements of the spatial weights matrix *W, w_ii_* is the weight when location *i* is in its own neighborhood, *ȳ* is the mean value of dependent variable, S^*^_i_ = ∑^*n*^_*j*=1_ *W*^2^_*ij*_ and *S* is the standard deviation of the dependent variable.

$$y_{i2}=\frac{\sum_{-=1}^{n}\sum_{j=1}^{n}{\left(y_{i}y_{j}\right)}^{2}}{n}-y_{i1}$$

A high value of the Getis-Ord G^*^_i_ statistic represents a group of high index values (hotspots), while a low value represents a low value of the index group. The hotspot analysis calculates Getis-Ord G^*^_i_ statistic for each feature in the data set.

**Moran's Scatter Plot and LISA Maps**: The Moran scatter plot is an illustration of the relationship between the values of the chosen attribute at each location and the average value of the same attribute at neighboring locations (27).

Clustered areas are categorized according to the pattern of characteristics in adjacent Kebeles. High/high (HH) areas are a set of Kebeles with high incidence RTAs surrounded by other Kebeles with high incidence of RTAs in univariate analysis. The same sense is applied to low/low (LL) set of Kebeles, where Kebeles with low characteristics are surrounded by other Kebels with low values for analyzed variables. When the inverse occurs, Kebeles with low incidence of RTA rate are surrounded by Kebeles with high incidence of RTA rate; LISA maps categorize them as low/high (LH) or high/low (HL) for the opposite pattern and identify significant spatial clusters throughout the study area, with high or low association values for traffic accident rates (27,28).

## Results

**Distribution of RTAs with respect to drivers' profile and road related factors**: The driver information was the key to model the risk factors related with road traffic accidents. As a result, shown in the Table 1, about 91.8 % of drivers involved in the traffic accident were male. Additionally, drivers below age category of below 18 years committed 160(38.4%) and those within 18–30 years committed 108(25.9%) of RTA.

**Table 1:** Distribution of RTAs with respect to drivers' profile and road related factors

Driver Profile	Category	Frequency	Percent
**Sex**	Male	383	91.8%
	Female	7	1.7%
	Unknown	27	6.5%
**Age Group**	<18	160	38.4%
	18–30	108	25.9%
	31–50	65	15.6%
	> 50	28	6.7%
	Unknown	56	13.4%
**Educational Level**	Lower than 10 Grade	109	26.2%
	10–12 Grade	131	31.4%
	Diploma and above	97	23.2%
	Unknown	80	19.2%
**Owner ship of formal driving**	Driver had no license	106	25.4%
**license**	Driver had formal license	243	58.27%
	Driver had informal license	48	11.5%
	Unknown	20	4.8%
**Drivers employment category**	Private employee	203	48.7%
	Owner	84	20.14%
	Gov't employee/NGO/Other	64	15.34%
	Unknown	66	15.8%
**Road segment**	Street	339	81.29%
	Curve	11	2.64%
	Sloppy	22	5.27%
	Intersection	22	5.28%
	Other (T Shape, Fixed Area, Roundabout)	17	4.07%
**Type of road at accident location**	Asphalt	342	82.01%
	Gravel	48	11.51%
	Coble stone	20	4.80%
	Fixed Area	1	0.24%

The numbers of RTAs committed by driver were found to have an association with their education level. For instance, out of the total 417 RTAs recorded in HCA during the study period, 109(26.2%) was recorded by drivers whose education level was below grade 10 while 131(31.4%) was recorded by drivers having education level between grade 10&12. Drivers with education level of diploma and above committed 23.2% of RTAs.

Different studies considered driving license ownership as a key determinant of studying RTA (11, 34, 35). As the result shown in Table 1, 106(25.4%) of RTA was reported from drivers who were driving without license while 48(11.5%) of RTA was reported from drivers had forged or non-equivalent driving license. From our study, majority (203(48.7%))of the total reported RTA was committed by private employed drivers while derivers who were driving their own vehicle committed about 84(48.7%) of the total reported RTAs.

Characterizing the distribution of RTAs at different sections of a road segment and type of road shade is useful in examining its clustering. Our result indicated that of the total 417 RTAs recorded during the study period, majority (339 (81.29%)) of RTAs were recorded on street roads having no junctions. Contrary, fewer amounts of traffic accidents was observed at curves, T shape junctions and roundabouts of the road segment.

Moreover, the results also indicated that of the total 417 recorded RTAs, 82.01% were on paved asphalt road while only 11.51% were recorded on gravel road. Relatively less percentage of RTAs were recorded on coble stone and fixed area roads (Table 1).

**Fig 1:**
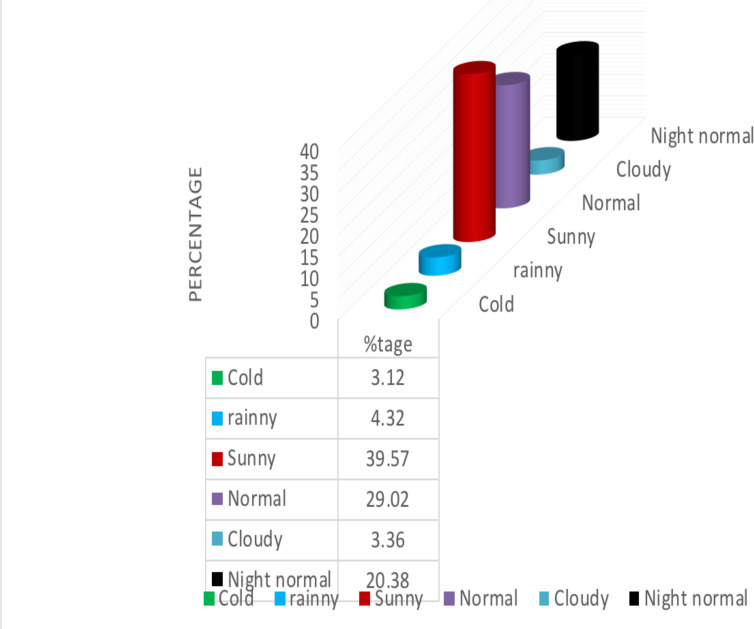
Percentage Distribution of RTAs by Weather Condition

**Distribution of traffic accident by weather condition**: The highest number of RTAs (39.57%) was recorded in sunny weather condition followed by normal weather condition during which 29.02% of RTA was recorded. In contrary, only about 3.12% and 3.36% of RTA was recorded during cold and cloudy weather conditions respectively (Figure 1).

**Fig 2:**
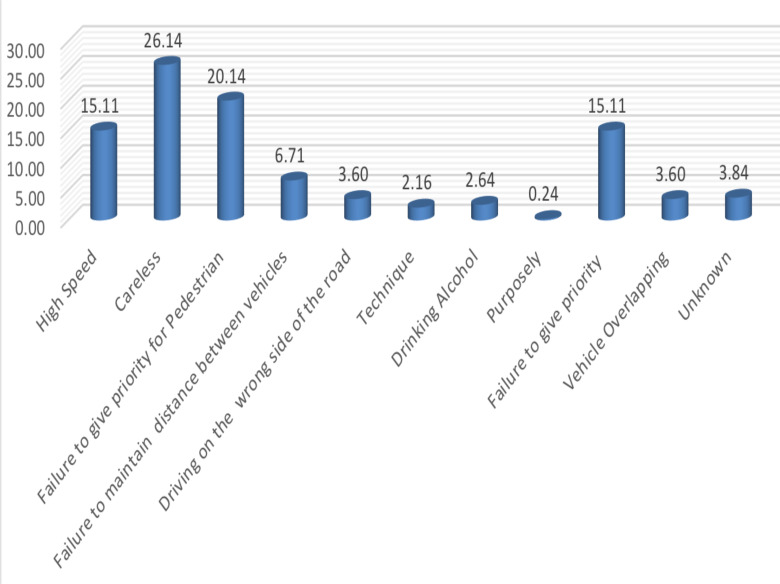
Percentage Distribution of RTAs by Reason of Accident at Scene

**Distribution of traffic accident by reason at accident scene**: Human factors such as rushing, negligence, speeding, alcohol etc are the most potent contributors to RTA in Ethiopia (2). In this regard, our finding shows that about 26.14% of drivers committed traffic accident due to careless driving habit while 20.14% of traffic accident was observed because of driver's failure to give priority for pedestrians. Also about 15.11% were observed due to high speed and/or driver failure to give priority to each other. The driver failure to maintain distance between vehicles was contributed about 6.71 % of traffic accidents whereas, driving on the wrong side of the road and overlapping were contributed about 3.6% of total accident (Figure 2).

**Exploratory Spatial Data Analysis (ESDA) results**: The Moran's I coefficient, being among the most widely implemented measures of spatial autocorrelation between neighbouring locations, provides some insights regarding the local spatial autocorrelation in traffic accident rate. In this sub-section, our focus is on its application to particular data analysis, the essential task being to seek for spatial dependence.

The annualized average incidence rate of RTAs per 1000 persons were calculated and tested for spatial dependence as depicted in Table 2. As the result, it reveals that average annual traffic accident was found tobe insignificant at 5% level (Moran's I=0.039, p-value=0.201). This evidences that there is no spatial autocorrelation of RTA rate across Kebeles in the study area. Hence, there is RTAs.

**Table 2:** Results of Moran's I Spatial Autocorrelation Test

Variables	Average Annual Incidence Rate	Moran's I Index	P-value
Total reported accidents	Accident per 1000 person	0.039	0.201
	Heavy Injury	-0.109	0.240
Type of injury	Death	-0.169	0.074
	Government employed	0.1645	0.255
Driver Category	Private employed	-0.0352	0.491
	Owner	0.126	0.070
	Motor Bike	0.0009	0.392
Vehicle Type	Public Transportation Vehicles	0.0145	0.263
	Private Use Vehicles	-0.034	0.473
Accident category	Vehicular Collision	0.0348	0.223
	Pedestrian Crash	0.047	0.213
Road type	Asphalt Road	0.108	0.074
	Gravel Road	0.1886	0.027*
Weather condition	Sunny	0.1878	0.028*
	Night Normal	0.044	0.215

* Significant at 5% level

In a similar fashion, except for gravel road type (Moran's I=0.1886, p-values= 0.027) and sunny weather condition (Moran's I=0.1878, p-value=0.028), Moran's I statistics found to be insignificant for all other variables. Thus, our hotspot analysis will focus on variables having significant Moran's I statistics which are gravel road type and sunny weather condition.

**Hotspot analysis**: The hotspots analysis was conducted particularly for RTAs recorded on gravel road type and sunny weather condition based on Moran's I test results obtained above. The hotspot calculation is based on Getis-Ord Gi* function (30).

**Fig 3:**
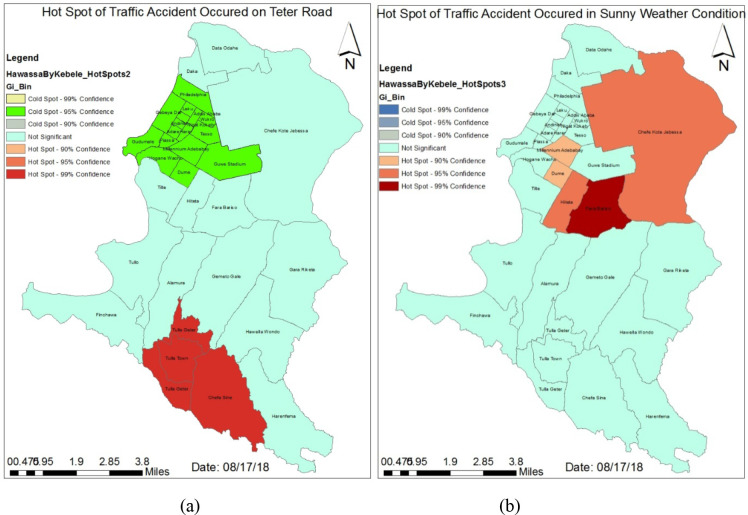
Hotspot Analysis of RTAs (a): Gravel Road (b): Sunny Weather Condition

As it can be observed in the Figure 3(a), Tulla town, Tulla Geter and Chefe Sine Kebeles were identified as hotspot areas for RTA occurring on gravel road type. On contrary, Philadelphia, Piassa, Kokeb Nigat, Addis Ababa, Millennium, Hogane Wacho, Gudumale, Guwe Stadium, Tesso, Wukro, Dume, Adare, Harer, Andinet, Leku and Gebeya Dar Kebeles were found to be cold spot areas of RTAs occurring on gravel road shade.

Similarly, Figure 3(b) revealed that Millennium Adebabay, Dume, Hiteta, Fara Bariso and Chefa Kote Jabessa Kebeles were hotspot areas for RTAs that occurred during sunny weather condition.

## Discussion

This study based on daily record of RTA data from traffic police department of HCA indicated that drivers under age category of 16–50 years of age (member of economically active population) contributed for 79.9% of RTAs the lion share being taken by adults within age group of 18–30. This signals that Ethiopia is losing significant amount of productive population due to RTA. This finding is in line with the findings of similar studies in Ethiopia by (2,3,6,12). A study conducted in Kenya by (31) also revealed that more than 75% of RTAs victims were economically productive young adults. The same result was reported using hospital-based study in India by (32).

Most of the studies conducted so far indicated that male drivers are the main victim of RTAs. Our study results indicate 91.8% of drivers involved in RTAs were males, while females share only 1.7% of the total reported RTA. This finding is in line with findings in previous studies in northern Ethiopia by (11) which reported that 99.6% of drivers involved in RTAs were male. It also agrees with finding of (10, 12) who reported that majority of RTA victims were males in Amhara and Southern National Regional States of Ethiopia. Moreover, this finding is consistent with the study conducted in Nigeria by (7), in Kenya by (31) and in Hong Kong by (33). Of course, in developing countries like Ethiopia, females are less engaged in outdoor activities and this may reduce their susceptibility to RTAs.

Our finding indicated that 37 % of drivers committed RTAs while they were driving without formal driving license. This is an implication that license ownership and driving experience are the most significant determinants of likelihood of committing RTA by drivers. On their studies of determinants of fatal car accident risk in Finote-Selam town in Ethiopia, (11) also reported that drivers with lack of sufficient driving experience caused to increase the chance of the occurrence of fatal accidents as compared to those with long years of driving experience. Similarly, a study by (34) also concluded that less experienced drivers were the most significant cause for RTA fatalities than experienced drivers. Another study by (35) also argued that drivers with no or lower driving experience usually involve in driving faster than the recommended speed limits.

Less educated drivers are responsible for lion share of RTAs in HCA. Out of 417 observed RTAs in the study area during the study period, 57.6% were committed by drivers whose education level was below diploma. This indicates that the likelihood of involvement in RTAs is higher for less educated drivers than that of educated drivers. A study conducted in northern Ethiopia by (11) also concluded that less educated drivers were more responsible for fatal injuries than educated ones. In line with this, a study conducted by (36) with an objective of identifying contributing factors for pedestrian crashes in Ethiopia also reported that less-educated drivers influence their chance of being involved in fatal RTAs. The same result was found by another study in Ethiopia by (3).

From our study, majority (48.7%) of the total reported RTAs were committed by private employed drivers. This evidences that private employed drivers were found to be at higher risk of committing RTA as compared with drivers who drive their own vehicle. Similar studies conducted in Ethiopia by (11, 35) also concluded that likelihood of fatal accidents is less for drivers who drive their own vehicle as compared to employed drivers.

Seasonality of occurrence of RTA is studied in different regions across the world. In HCA, 39.57% of the RTAs occurred in sunny weather condition. This may be related with traffic police controllers failing to impose traffic rules during sunny days. The finding opposes previous findings in Amhara region in Northern Ethiopia by (2) which reported that 91% of road traffic crushes occurred in rainy season. It also opposes findings in India by (37) which reported that highest numbers (32.3%) of RTAs were observed during heavy rainy season.

Human factors such as rushing, negligence, speeding, alcohol etc are the most potent contributors to RTA (21,35). In this regard, our finding shows that about 26.14% of drivers committed RTA due to careless driving habit while 20.14% of RTA was observed because of drivers' failure to give priority for pedestrians. In addition, about 15.11% were observed due to high speed and/or driver failure to give priority to each other. Previous study in Amhara Regional State in Ethiopia by (2) reported that 31.5% of RTAs were due to speed factor. A study in United States of America by (21) also reported that 31% of the total crashes recorded in Baltimore city between 2009 GC and 2013 GC were due to distracted driving behavior while speeding was recorded as a reason for 6% of crashes. A study by (37) in India also indicated that 95.4% of RTAs were accounted by human characteristics. In Ghana, the speed factor alone accounted for more than 50% of RTA (38). From these evidences, it can be concluded that reducing vehicle speed can be the most effective interventions to mitigate RTAs.

Furthermore, characterization of traffic accident distribution at different sections of a road junction, type of road shade and condition at accident scene was useful to study its clustering across regions of interest (29, 39). The current study reveals that, large number (81.29%) of accidents occurred on street roads having no junctions. This result might be related with higher percentage of drivers committed accidents with high speed and careless driving habit. Additionally, about 82.01% of traffic accidents were recorded on asphalt road while 11.51% of RTAs were recorded on gravel road. Identifying road traffic accidents hotspots is a key role to make basic strategies and polies to reduce the densest locations of accident (20–23,39). The spatial autocorrelation test of clustering was conducted through Moran's I index for average annual traffic accidents incidence rate per 1000 persons. As a result, the Moran's I index for average annual RTA was found to be insignificant at 5% level. It shows there was no clustering of RTA across Kebeles of HCA. This result opposes previous findings on which spatial autocorrelation was detected for RTA data (20–23,29). Thus, RTA follows random distribution rather than exhibiting spatial clustering in HCA.

The tests of spatial autocorrelation for traffic accident occurred on gravel road and in the sunny weather condition were found to be significant at 5% level (Moran's I =0.1886 and 0.1878 respectively). Therefore, there was a spatial dependency of RTA across Kebeles of HCA for accidents recorded on gravel road and during sunny weather conditions. This result was supported by results obtained in the study conducted in India which indicated that the accident hotspots as well as cold spots are clustered around specific sectors with isolated highs and lows and show spatial variation among the datasets (29).

The analysis of hotspots was conducted through Getis-Ord Gi* statistics (30) particularly for RTAs occurred on gravel road shade and in sunny weather condition (Figure 3). The study reveals that Tullatown, Tulla Geter and Chefe Sine were identified as hotspot Kebeles for RTA occurred on gravel road. On contrary, Philadelphia, Piassa, Kokeb Nigat, Addis Ababa, Millenium, Hogane Wacho, Gudumale, Guwe Stadium, Tesso, Wukro, Dume, Adare, Harer, Andinet, Leku and Gebeya Dar Kebeles were found to be cold spot areas for RTA occurred on gravel road.

Similarly, Millenium Adebabay, Dume, Hiteta, Fara Bariso and Chefa Kote Jabessa Kebeles were identified as hotspot areas for RTA occurred in sunny weather condition.

Lack of sufficient data on several important predictors, if included in the analysis, may affect RTA is the main limitation of this study. Because of under-reporting, the number of records in the study may not be adequate. Further spatio-temporal modelling is recommended to investigate temporal variation across clustering of road traffic accidents.

In conclusion, fatalities and injuries related to RTA are significantly high in HCA and it most affects economically productive members of the society. Less educated male drivers who were hired by private employees are at increased risk of RTAs. Special attention is needed for private employed, less educated, younger and un-licensed drivers. Government and concerned stake holders are recommended to revise rules regarding age limit and education level while giving driving license.

Much emphasis must be given at straight road to minimize the occurrence of RTA. Scaling up gravel road to asphalt road is necessary for three hotspot Kebeles namely, Tulla town, Tulla Geter and Chefe. RTA should be considered as a priority public health problem in Ethiopia, particularly in HCA.
